# Quantifying individual-level heterogeneity in infectiousness and susceptibility through household studies

**DOI:** 10.1016/j.epidem.2023.100710

**Published:** 2023-07-22

**Authors:** Thayer L. Anderson, Anjalika Nande, Carter Merenstein, Brinkley Raynor, Anisha Oommen, Brendan J. Kelly, Michael Z. Levy, Alison L. Hill

**Affiliations:** a Institute for Computational Medicine, Johns Hopkins University, Baltimore, MD 21218, United States of America; b Department of Microbiology, Perelman School of Medicine, University of Pennsylvania, Philadelphia, PA 19104, United States of America; c Department of Biostatistics, Epidemiology, & Informatics, Perelman School of Medicine, University of Pennsylvania, Philadelphia, PA 19104, United States of America; d Department of Biomedical Engineering, Johns Hopkins University, Baltimore, MD 21218, United States of America; e Division of Infectious Diseases, Department of Medicine, Perelman School of Medicine, University of Pennsylvania, Philadelphia, PA 19104, United States of America

**Keywords:** Household transmission, Superspreading, Heterogeneity, Attack rate, COVID-19

## Abstract

The spread of SARS-CoV-2, like that of many other pathogens, is governed by heterogeneity. “Superspreading,” or “over-dispersion,” is an important factor in transmission, yet it is hard to quantify. Estimates from contact tracing data are prone to potential biases due to the increased likelihood of detecting large clusters of cases, and may reflect variation in contact behavior more than biological heterogeneity. In contrast, the average number of secondary infections per contact is routinely estimated from household surveys, and these studies can minimize biases by testing all members of a household. However, the models used to analyze household transmission data typically assume that infectiousness and susceptibility are the same for all individuals or vary only with predetermined traits such as age. Here we develop and apply a combined forward simulation and inference method to quantify the degree of inter-individual variation in both infectiousness and susceptibility from observations of the distribution of infections in household surveys. First, analyzing simulated data, we show our method can reliably ascertain the presence, type, and amount of these heterogeneities given data from a sufficiently large sample of households. We then analyze a collection of household studies of COVID-19 from diverse settings around the world, and find strong evidence for large heterogeneity in both the infectiousness and susceptibility of individuals. Our results also provide a framework to improve the design of studies to evaluate household interventions in the presence of realistic heterogeneity between individuals.

## 1. Introduction

In the early months of the COVID-19 pandemic, contact tracing efforts revealed that superspreading events — when a single infected individual transmits to a large number of secondary individuals — play an important role in the spread of SARS-CoV-2 (Mizumoto et al., 2019; Hodcroft, 2020; Jang et al., 2020; Jones and Maxouris, 2020; Holcombe, 2020; Althouse et al., 2020). According to a systematic review, 21/26 studies quantifying SARS-CoV-2 superspreading reported significant over-dispersion in transmission and found that between 1% to 38% of individuals were responsible for 80% of secondary infections (with estimates for the negative binomial dispersion parameter k ranging from 0.01 to 0.72) (Wang et al., 2021). Superspreading has also been recognized as a key feature of transmission dynamics for a wide variety of other infections including SARS (Lloyd-Smith et al., 2005; Adam et al., 2020), MERS (Kucharski and Althaus, 2015), smallpox (Lloyd-Smith et al., 2005), Ebola (Lau et al., 2017), tuberculosis (Smith et al., 2022; Ypma et al., 2013; Melsew et al., 2019), and HIV (Galvani and May, 2005).

These dynamics highlight the limitations of summarizing transmission patterns using only average quantities, like the basic reproductive number, *R*_0_ (Hébert-Dufresne et al., 2020). More generally, individuals may vary in both their infectiousness (propensity for onward transmission if infected) and susceptibility (propensity to become infected given exposure). This heterogeneity is caused by a mix of host behavior, host-pathogen interactions within the body, and environmental factors (MacDonald et al., 2000; Bunyavanich et al., 2020; Tobian et al., 2010; Gray, 2019; Bajwa, 2019; K.A. and A.P., 2017; Aw et al., 2007; Dixon et al., 2011; Favalli et al., 2020; Gomes et al., 2014; Bansal et al., 2007; Chen et al., 2021; Memoli et al., 2015; Watanabe et al., 2010; Quinn et al., 2000; Corey et al., 2004; Cohen et al., 2016; Chase-Topping et al., 2008; Lloyd-Smith et al., 2005; Plowright et al., 2017; Lau et al., 2017). For example, variation in infectiousness can stem from heterogeneity in the number, type, and intensity of an index individual’s contacts (Bansal et al., 2007); heterogeneity in pathogen shedding in respiratory, genital or fecal excretions (Chen et al., 2021; Memoli et al., 2015; Watanabe et al., 2010; Quinn et al., 2000; Corey et al., 2004; Cohen et al., 2016; Chase-Topping et al., 2008); infectious period duration (Lloyd-Smith et al., 2005; Plowright et al., 2017; Lau et al., 2017); and pathogen survival outside the host body (Tang, 2009; Vasickova et al., 2010; Suleyman et al., 2018; Marzoli et al., 2021). Host susceptibility has been observed to vary based on immune status due to genetics (MacDonald et al., 2000), age (Aw et al., 2007), disease (e.g., AIDS CDC, 2022), medication (e.g., Dixon et al. 2011, Favalli et al. 2020), temperature (e.g., Foxman et al. 2016), or previous exposure due to infection or vaccination (Gomes et al., 2014), as well as other phenotypes (e.g., Bunyavanich et al. 2020, Bajwa 2019, K.A. and A.P. 2017, Tobian et al. 2010, Gray 2019). Models have demonstrated that variation in infectiousness plays a crucial role in determining the extinction probability of an outbreak following introduction into a new population (Lloyd-Smith et al., 2005; Miller, 2007), the final size of an epidemic (Hébert-Dufresne et al., 2020), the efficacy of contact tracing (Klinkenberg et al., 2006), the rate of emergence of pathogen variants (Gómez-Carballa et al., 2021), and the optimal allocation of measures to prevent infection or reduce transmission (Medlock and Galvani, 2009). Variation in host susceptibility affects the epidemic growth rate, the herd immunity threshold and the final epidemic size (Gomes et al., 2014; Rose et al., 2021; Dwyer et al., 1997; Miller, 2007), and provides opportunities for targeting preventative measures such as vaccination (Medlock and Galvani, 2009; Wallinga et al., 2010; Preciado et al., 2013; Fitzpatrick and Galvani, 2021; Buckner et al., 2021; Bubar et al., 2021; Matrajt et al., 2021).

While characterizing variation in infectiousness and susceptibility is a critical part of designing accurate mathematical models and effective public health interventions, it is difficult to quantify this variation in practice. Population-level epidemic growth patterns that are used to infer *R*_0_ (e.g., Cauchemez et al. 2006, White and Pagano 2008) can rarely uniquely identify variation among individuals. Contact tracing studies are the most common tool used to estimate the degree of variation in infectiousness, but they suffer from possible biases. The size of chains of infection may be underestimated when relying on individuals naming known contacts, but at the same time, superspreading events may be more likely to be detected by surveillance efforts than “normal” chains of infection due to the number of individuals they involve (Wang et al., 2021; Endo et al., 2020). Lloyd-Smith found that joint estimation of *R*_0_ and the degree of variation in infectiousness was particularly difficult with certain types of censoring present in outbreak data (Lloyd-Smith, 2007). Variation caused by host behavior like travel patterns — as opposed to biological or environmental causes — might have an outsized effect on these studies. Public policy measures such as mask mandates can increase the fraction of transmission stemming from superspreading events, as those events are likely to involve greater risk and fewer precautions (Endo et al., 2020). Mild cases of disease can involve lower viral load and therefore lower probability of onwards transmission but also a lower probability of detection, which may further bias estimates of heterogeneity (e.g. Zheng et al. 2020).

Household transmission studies are a common tool in infectious disease epidemiology that sidestep many of these possible biases. Households are a context where it is known — or can be reasonably assumed — that all individuals are in close contact with each other and where infection status can be ascertained for the entire group. However, household studies typically calculate only the *average* probability of infection per contact, known as the secondary attack risk (SAR). Simple estimates of the SAR assume all infected secondary contacts are caused directly by the primary case (reviewed in Sharker and Kenah 2021, example in Papenburg et al. 2009), while other studies account for unknown transmission pathways over multiple generations of infections using the ‘Reed-Frost’ model (also called the ‘chain binomial’ model) (Abbey, 1952) or statistical methods to infer missing infection times using data-augmentation techniques (Cauchemez et al., 2009, 2004). When household studies evaluate heterogeneity at all, it is commonly by stratifying cases by a pre-specified individual trait (like host age) (Tsang et al., 2016). Reed-Frost models have been extended to “multitype” versions in which distinct classes of individuals have different infectivities (Pellis et al., 2008; Scalia-Tomba, 1986) and “collective” versions where infectiousness varies continuously across individuals (Pellis et al., 2008; Picard and Lefevre, 1990; Lefe’vre and Utev, 1999), and have been used to estimate jointly the SAR and variation in infectiousness for the 1918 influenza pandemic (Fraser et al., 1918), 2009 “swine flu” (H1N1) (Nishiura et al., 2009), and COVID-19 (Bi et al., 2021; Toth et al., 2021). A stochastic, discrete-generation chain binomial model has the benefit of admitting analytic solutions for the outbreak size distribution in a finite, well-mixed population like a household, and is often mathematically equivalent to continuous time models (Ludwig, 1975; Pellis et al., 2008). However, its use requires the absence of any parameter dependence on calendar time and is therefore unsuitable in circumstances where interventions such as availability of vaccines/therapeutics, out-of-home isolation, or changes in household composition play a substantial role in infection dynamics (e.g. Nande et al. 2021).

In this study we develop a method to simultaneously estimate the SAR and the degree of heterogeneity in infectiousness and susceptibility from household transmission studies. Our method can use arbitrary models of disease dynamics and arbitrary distributions of individual-level traits. For emerging pathogens, knowledge of the individual traits that modify infection risk is often lacking (Bansal et al., 2007), and our approach avoids *a priori* stratification by modeling the individual differences in a continuous — rather than a discrete — way. Our approach combines direct model simulation with formal likelihood-based parameter inference. We can consistently estimate key epidemiological parameters with modest computational resources, with the precision of parameter estimates and the power to identify the impact of interventions increasing with both study size and the inclusion of households with more than two individuals. We find that ignoring heterogeneities, when they exist, can lead to biased estimates of the SAR. Applying this method to multiple existing households studies of COVID-19 in diverse settings, we quantify both types of transmission heterogeneity. We hypothesize that studying the variability of pathogen spread within households can limit the sources of measured heterogeneity to those sources that are tied most closely to host-pathogen interactions and biology, which may be more generalizable across settings.

## 2. Methods

### 2.1. Model of transmission within households

**Fig. 1.**
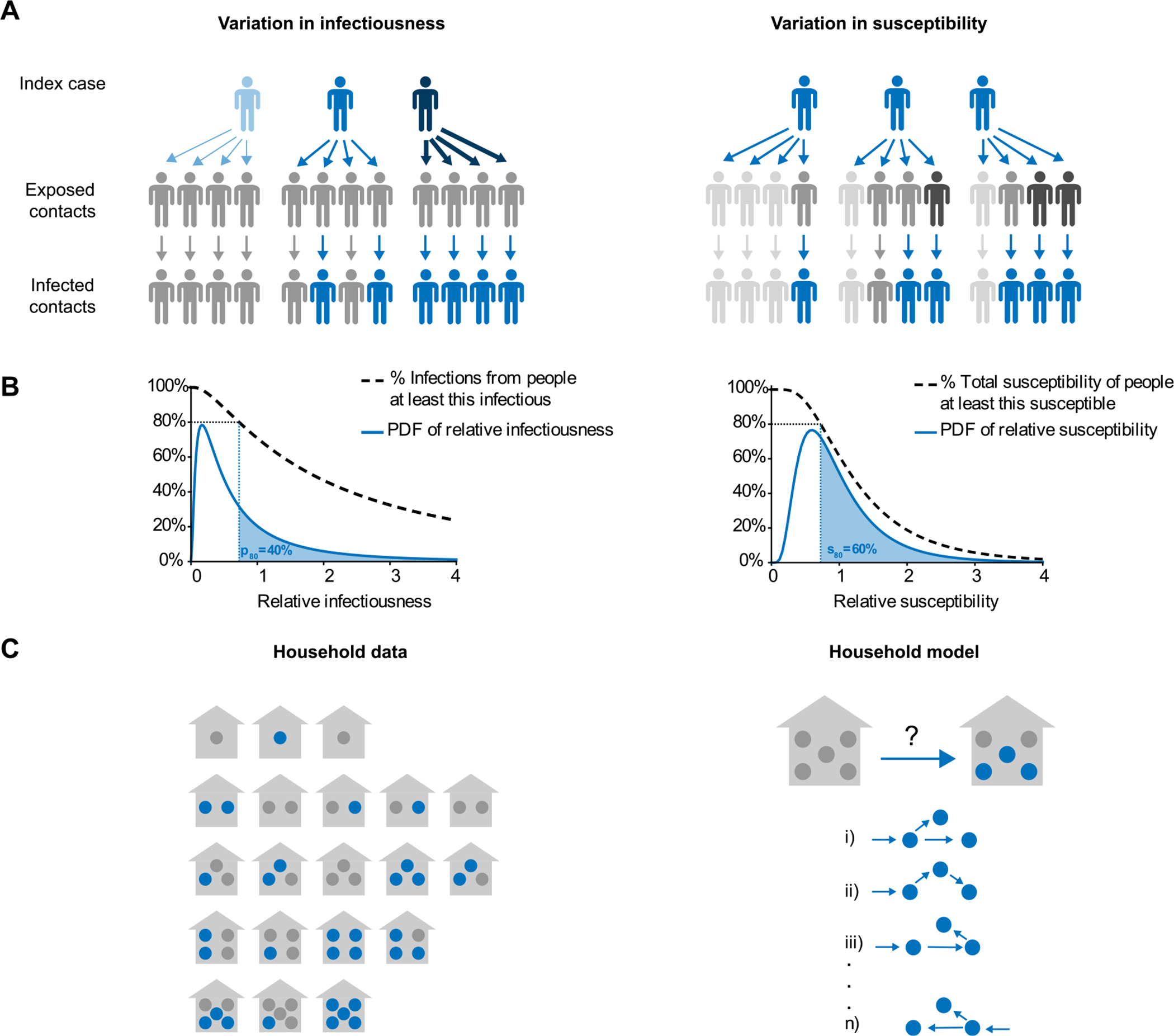
Model schematic for individual-level heterogeneity and household transmission studies. (A) The effect of variation in infectiousness (left) and susceptibility (right) on an imagined chain of infections across two generations of spread. Gray = uninfected, Blue = infected. Darker color = more susceptible/infectious. (B) Left: Probability density function for relative individual infectiousness and representation of relationship between $p_{80}$ (shaded area), relative infectiousness (*x*-axis), and the fraction of infections from people at least that infectious (*y*-axis). Right: Probability density for relative susceptibility, and representation of relationship between $s_{80}$ (shaded area), relative susceptibility (*x*-axis), and fraction of total susceptibility held by people at least that susceptible (*y*-axis). (C) Left: Example dataset from a household transmission study comprised of only the distribution of final household outbreak sizes. Right: Possible chains of infection, simulated by the model (but unknown for the data), that might give rise to the final observed outbreak size in a household.

The focus of our model is a population in which individuals are divided up into households of different sizes (Fig. 1C). Each household is assumed to be a well-mixed population with a single introduction of infection. We consider diseases with finite infectious periods (e.g., diseases that lead to eventual recovery and immunity) and assume the outbreak within each household is complete by the end of the study period. These assumptions are valid when the study period is long relative to the infectious period, and when an individual’s risk of transmission from the community is much smaller than the risk of transmission from within a household, given at least one infected household contact. Our direct simulation method allows for relaxation of these assumptions if desired. We track the number of individuals within each household who were ever infected (final epidemic size) and assume no further information is available about the order or timing of infection.

When an infected individual and a susceptible individual are in the same household, the average probability per unit time that infection passes between them is *β*, but the specific rate varies between pairs of individuals due to the heterogeneity in their per-contact infectiousness and per-contact susceptibility. We summarize the transmission potential of an infection with the household secondary attack risk (SAR), defined as the average probability of infection per household contact caused by a single initial introduction of the pathogen (i.e., not taking into account chains of infection beyond the initial → secondary). The mathematical relationship between the SAR and *β* depends on the nature of the variation in infectiousness and susceptibility across individuals, as well as on the distribution of the duration of infectious periods (see Supplementary Methods).

Our inference framework allows for the specification of any model of transmission within households. For simplicity and for consistency with standard methods for the simulation of COVID-19 dynamics, we choose to model transmission with a stochastic SEIR (susceptible, exposed, infectious, recovered) process. We assume that the times spent in the exposed and infectious states follow lognormal distributions, a long-tailed distribution chosen because it simplifies the calculation of expected secondary infections (see Supplementary Methods). For the latent period, we choose parameters of the distribution such that the average length is 3.5±2.5 days, and for the infectious period, we choose them so that the length is 6±2.5 days (consistent with Zhao et al. 2021 and references therein).

### 2.2. Modeling heterogeneity in infectiousness and susceptibility

Under our model, each individual in a household is assigned two traits: one that determines their relative per-contact infectiousness and one that determines their relative per-contact susceptibility (Fig. 1). In the population, these traits are independent and continuously-valued random variables. We ignore the differing underlying causes of this variability (e.g. host genetics vs. behavior vs. viral factors) and focus only on the net heterogeneity. This modeling approach is similar to including random effects in regression models or frailties in survival models. Beyond these traits, individuals are identical and we assume the distributions of traits are independent of factors such as age or biological sex. The most important consequence of heterogeneity in infectiousness is over-dispersion in the expected number of secondary infections caused by each individual. As a result, it is common to quantify the heterogeneity in infectiousness in terms of the distribution of expected infections, for example with the “dispersion parameter” k of the negative binomial distribution (Lloyd-Smith et al., 2005). We take an alternative approach (Galvani and May, 2005; Kremer et al., 2021) and quantify heterogeneity with $p_{80}$, defined as the (smallest) fraction of individuals responsible for 80% of secondary infections on average (Fig. 1). This fraction is calculated in reference to the hypothetical offspring distribution if each individual were placed in an infinite well-mixed population, and thus is the “expected” proportion of transmission (Lloyd-Smith et al., 2005), as opposed to the “observed” proportion that includes inherent stochasticity (see e.g., Endo et al. 2020). When $p_{80}=80\%$, each individual is equally infectious. For $p_{80}<80\%$, a smaller fraction of individuals is responsible for a greater portion of secondary infections (i.e., the distribution is over-dispersed Endo et al., 2020; Kremer et al., 2021). We model the distribution of relative infectiousness in the population as a lognormal random variable with natural-scale mean of 1, and we numerically solve for the variance given a desired value of $p_{80}$. For a full description of this approach, see the Supplemental Methods.

We describe variation in relative susceptibility through the quantity $s_{80}$, the smallest fraction of the population that must be infected in order to reduce the total remaining susceptibility by 80% of its initial total (and therefore reduced *to* 20% of its initial total). This approach is based on the fact that as an infection spreads through a population with heterogeneous susceptibilities, the average susceptibility in the uninfected population decreases over time as more susceptible individuals are infected first with greater probability. When $s_{80}=80\%$, all individuals are equally susceptible. If $s_{80}=20\%$, then the most susceptible 20% of individuals are responsible for 80% of the total susceptibility. If they were all infected, the average susceptibility of uninfected individuals would be 20% of its initial mean. We model susceptibility variation as another lognormal random variable with mean 1, and we calculate the variance based on the specified $s_{80}$. When infection is first introduced into a household, the index individual is randomly assigned with probability in proportion to the susceptibility of each household member.

### 2.3. Parameter inference

To make inferences, we calculate the likelihood of observing a particular dataset under the proposed model of household transmission for each possible set of model parameters (θ), and then find the set of parameters that maximizes this likelihood function. The datasets we consider consist of the number of infections in each household in a cohort, where households can be grouped by number of members (Fig. 1C). Formally, the data is a set of $y_{n,k}$, the total number of households of size n observed to have k infections.

We calculate the probability $p_{M}(n,k,\theta )$ under our model M that a single household of size n observes k infections after a single introduction by simulating a large volume of households of size n forward in time from their initial state and treating the frequency of occurrences for the different k’s as the probability of observing that many infections. Then, the likelihood of observing the full dataset given the model, ℒ(Y,θ), is given by the multinomial:

(1) $$ℒ\left(\left\{y_{n,k}\right\}|\theta \right)=\prod_{n=2}^{n_{\text{max}}}\prod_{k=1}^{n}p_{M}{\left(n,k,\theta \right)}^{y_{n,k}}$$

By varying the parameters θ and repeating forward simulations of our model at each chosen value of θ, we construct a likelihood surface. The maximum likelihood estimate of the parameter values (MLE) is the position of θ that produces the greatest likelihood of all tested combinations of parameter values. The likelihood surface can be viewed as a (discrete) probability density function of a posterior probability distribution in a Bayesian framework by making the assumption of uniform priors on all parameters and then normalizing. To calculate 95% confidence intervals (equivalently, credible intervals), we rank each tested θ value from most probable to least probable, include θ values in the interval in order of descending probability until at least 95% of the overall probability is represented, and then letting the extreme values for each parameter be the bounds of the confidence interval.

In this paper the unknown parameters that we infer are (1) $p_{80}$, (2) $s_{80}$, and (3) the average transmission rate β — although we actually infer a transformation of β, SAR, the average secondary attack risk in the population (Supplemental Methods). For inference on real data from COVID-19 outbreaks and for benchmarking tests on simulated data, we assumed that the other model parameters — the duration of the latent period and infectious period — are known exactly (i.e., not inferred).

More details about our simulation-based approach to calculate this likelihood surface, find the most likely θ, and calculate uncertainty bounds are also described in the Supplemental Methods. All of the code and data required to reproduce the results in this paper is openly available via GitHub: https://github.com/tanderson11/householdheterogeneity.

## 3. Results

### 3.1. The effect of heterogeneity on household outbreaks

**Fig. 2.**
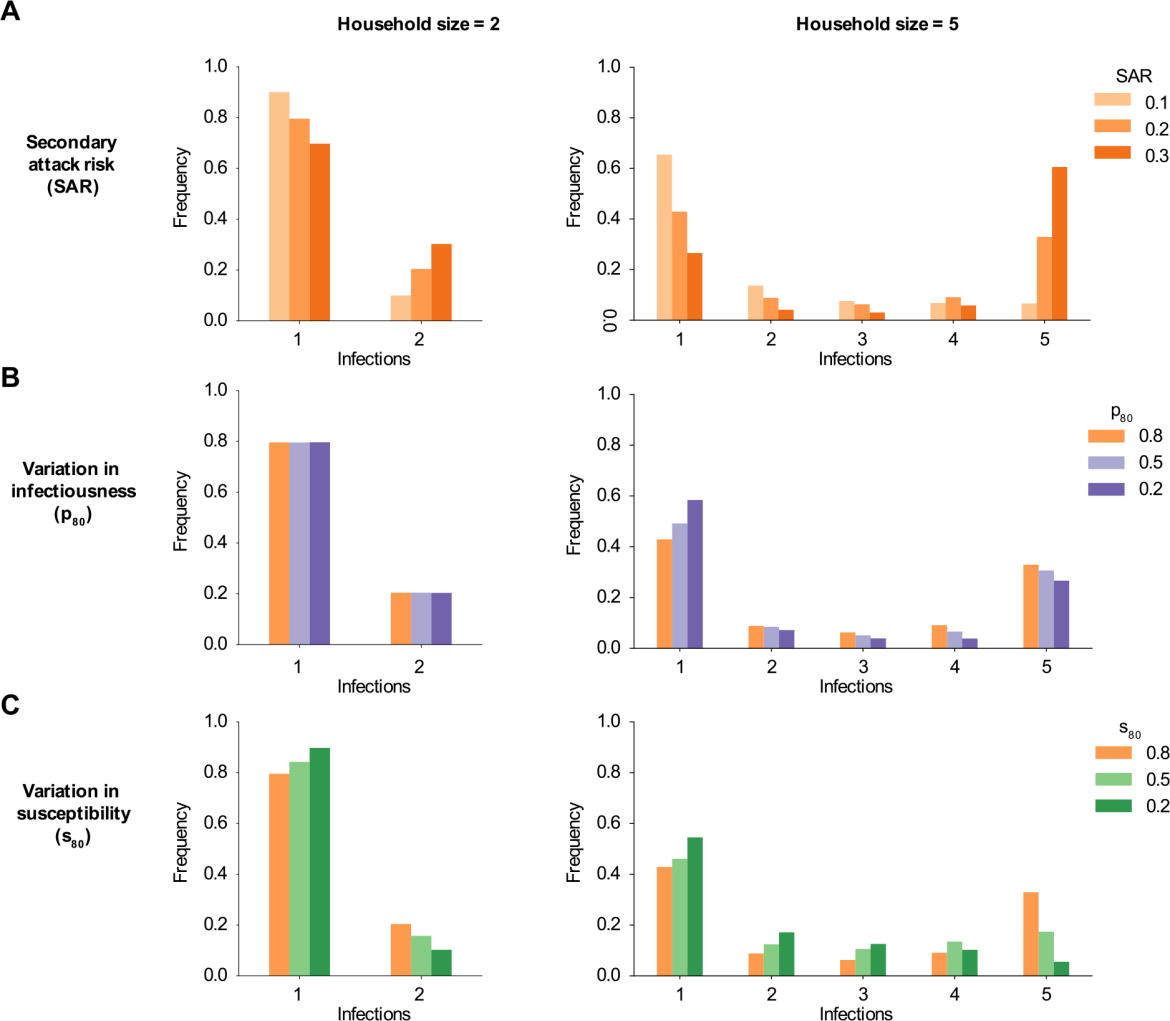
The effect of heterogeneity in infectiousness and susceptibility on the distribution of infections in a household outbreak. Histograms of simulated infection outcomes (including index case) in households of different sizes after a single introduction of a pathogen. *x*-axis: number of infections in household. *y*-axis: frequency at which that number of infections is observed in all households with at least one infection. (A): Comparison of infections in households of size 2 (left column) and size 5 (right column) for different values of the average secondary attack risk (SAR). (B) Simulated infections with SAR=20% and different amounts of infectiousness variation (more when $p_{80}$ is smaller: darker purple). (C): Simulated infections for SAR=20% and different amounts of susceptibility variation (more when $s_{80}$ is smaller: darker green).

Simulating the spread of infection in households, we found that the average risk to a susceptible contact (SAR), the amount of inter-individual variation in infectiousness ($p_{80}$), and the amount of inter-individual variation in susceptibility ($s_{80}$) each affect the final size of household outbreaks in distinct ways (Fig. 2). Predictably, we observed that a greater SAR increases the frequency with which an outbreak caused by a single introduction will spread to other individuals in a household and consequently lowers the within-household extinction probability (i.e., the frequency of outbreaks of size 1) and increases the frequency of larger outbreaks (Fig. 2A).

Variation in infectiousness (superspreading) increases “feast or famine” dynamics: outbreaks are more likely to go extinct and more likely to infect all household members provided that they do not go extinct (Fig. 2B).

Susceptibility variation, in contrast, increases the probability of extinction but also concentrates the distribution of final sizes at intermediate numbers of infections and reduces the probability that the entire household is infected (Fig. 2C).

As an example, in a household of size 5 with SAR=20% and no variation in infectiousness or susceptibility, an outbreak results in a single infection 43% of the time, 3 infections 6% of the time, and 5 infections 33% of the time. In the same household when the most infectious 20% of individuals are responsible for 80% of secondary infections $\left(p_{80}=20\%\right)$, the frequency of outbreaks of size 1, 3, and 5 becomes 58%, 4%, and 27%. Alternatively, if there is variation in susceptibility such that the most susceptible 20% of individuals hold 80% of the population’s total susceptibility $\left(s_{80}=20\%\right)$, the frequency of outbreaks of size 1, 3, and 5 becomes 55%, 13%, and 5%.

In households of size 2, the effects of variation in infectiousness and susceptibility are more difficult to discern. The SAR alone determines the *average* risk to the second household member. Infectiousness variation does not affect outcomes (Fig. 2b). Since a more susceptible individual is proportionally more likely to be the site of introduction, other household members have on average lower susceptibility and a reduced risk of secondary infection. Susceptibility variation therefore still affects the outbreak size in households of size 2 for a fixed average infection probability (SAR).

These distinct patterns in the final size distribution of household outbreaks suggest that it is possible to infer the SAR and the heterogeneities simultaneously from data.

### 3.2. Inferring heterogeneity parameters from household outbreak sizes

**Fig. 3.**
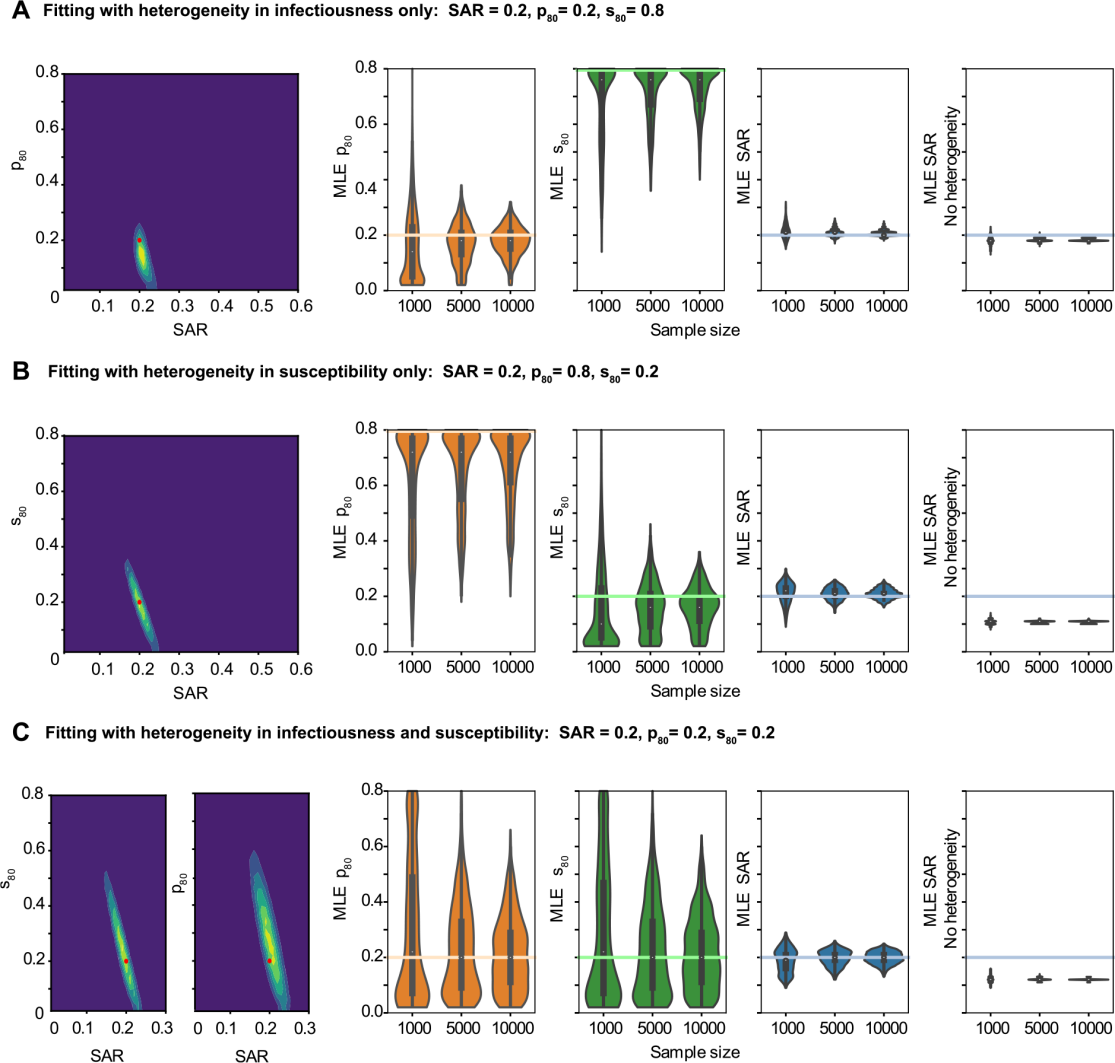
Maximum likelihood estimates for SAR and degree of heterogeneity on simulated data in a variety of parameter regimes. Simulated datasets were generated with 1000, 5000, or 10,000 individuals divided among households with a household size distribution taken from the United States census. (A) Simulated data includes only variation in infectiousness ($p_{80}$). (B) Simulated data includes only variation in susceptibility ($s_{80}$). (C) Simulated data includes variation in both infectiousness and susceptibility. For (A)-(C), the left panel shows the likelihood surface describing the probability of the model parameters given a simulated dataset (yellow is more likely, blue is less likely). This two dimensional surface marginalizes over the third, not shown, parameter. The red dot shows the true input parameter value. The three middle panels show the distribution of maximum likelihood estimates (MLEs) for the three parameters of interest — the secondary attack risk (SAR), the variation in infectiousness ($p_{80}$), and the variation in susceptibility ($s_{80}$). For each panel, the colored horizontal line is the actual value of the parameter used to generate the simulated data, and the white dot is the mean of the individual MLEs. The rightmost panel shows the MLE for the SAR if heterogeneities are not included in the model inference framework. Parameters were estimated for 1,750 different simulated datasets. Other model parameters were fixed as described in the Methods.

Before trying to infer heterogeneity from real-world data collected in studies of household outbreaks, we first confirmed that our inference method could recover the input parameters used to generate simulated data. We evaluated scenarios where only one kind of heterogeneity was present, as well as scenarios where there was heterogeneity in both traits (Fig. 3). We found that the SAR can be accurately and precisely inferred regardless of the kinds and amount of heterogeneity present, provided that the model is correctly specified. For simulated datasets of 5000 individuals with high heterogeneity in both traits and an SAR of 20%, the maximum likelihood estimate (MLE) of the SAR was in the range (15%, 25%) 95% of the time (Fig. 3C). For any individual simulated dataset with 5000 individuals, the uncertainty in the estimated SAR was around ±7% points. However, when the model used for inference does not incorporate heterogeneity while it is present in the data-generating dynamics, significant bias is introduced into the estimate of the SAR — particularly when a high degree of susceptibility variation is present. For the simulated datasets with SAR=20% described above, the MLE estimates with a misspecified model were centered on ≈19% in the absence of susceptibility variation and ≈11% in its presence.

Unbiased estimates of the degree of heterogeneity in infectiousness ($p_{80}$) or susceptibility ($s_{80}$) can also be inferred from simulated data, albeit with significantly more uncertainty even for larger sample sizes (Fig. 3A, B). For example, for simulated datasets of 5000 individuals, a value of $p_{80}=20\%$ could be inferred to within (2%, 38%) in 95% of cases (to within (0%, 52%) if both heterogeneities are present) and $s_{80}=20\%$ to within (0%, 40%) in 95% of cases (or (0%, 54%) if both heterogeneities are present).

For smaller sample sizes, the presence or absence of each type of heterogeneity can be determined reliably even if their exact value cannot be inferred. When one kind of heterogeneity was absent from simulated data ($s_{80}=80$ or $p_{80}=80\%$) and the other was present, the best estimates for the absent heterogeneity reliably suggested that at most slight variation was present. For samples of 1000 individuals where $p_{80}=80\%$, 65% of estimates for that parameter fell in the range $60\%\leq p_{80}\leq 80\%$. For $s_{80}=80\%$, 75% of the estimates fell in that range. The maximum likelihood estimates for the parameter describing the heterogeneity that was in fact present even more reliably indicated moderate to extreme heterogeneity. For $p_{80}=20\%$ and $s_{80}=80\%$, the best estimate for $p_{80}$ was less than 40% in 94% of cases. For $p_{80}=80\%$ and $s_{80}=20\%$, $s_{80}$ was less than 40% in 96% of estimates.

When both kinds of heterogeneities are present (Fig. 3C), the necessary sample size for a precise measurement of all three parameters is large (upwards of thousands of households). Some of the uncertainty in the parameter governing heterogeneities in transmission dynamics comes from a degree of mutual non-identifiability between these parameters and the SAR in smaller households (lack of identifiability can be seen from the ridged likelihood surfaces in Fig. 3B and C; an examination of small households is made in Supp Figure S1).

Overall, these analyses suggest that reasonable estimates of the amount of inter-individual variation in susceptibility and infectiousness can be made given a large enough cohort of households, and that even with smaller sample sizes the presence versus absence of these heterogeneities can be inferred.

### 3.3. Quantifying heterogeneities in household studies of COVID-19

**Fig. 4.**
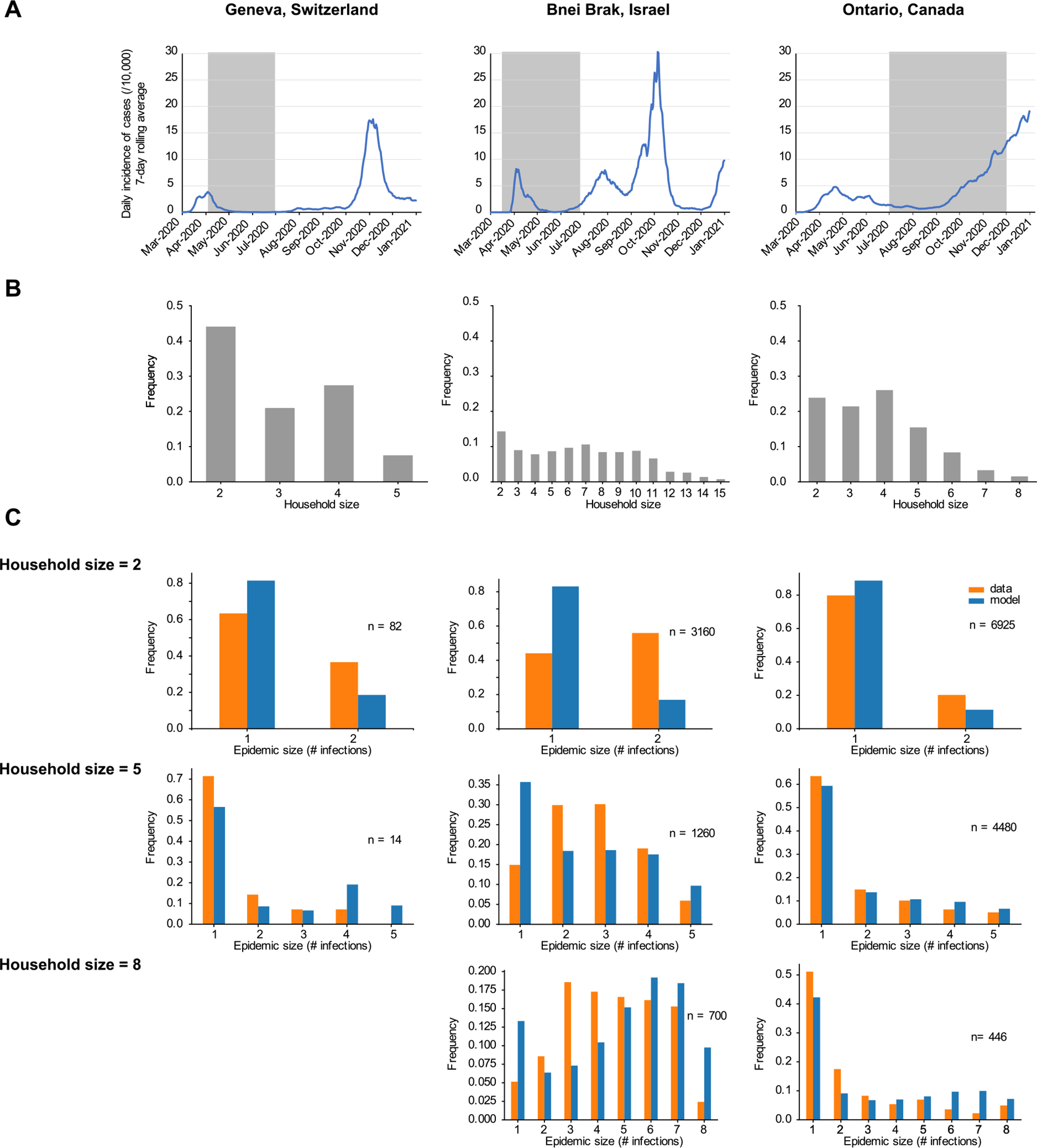
Estimating transmission heterogeneity parameters from household studies of COVID-19. (A) Timing of study (highlighted region) compared to incidence in the region throughout 2020 (daily cases per 10,000 inhabitants, 7-day rolling average) (Bi et al., 2021; Open Data of the Canton of Zürich, 2022; Dattner et al., 2021; Ministry of Health of Israel, 2022; Tibebu et al., 2021; Public Health Ontario, 2022). (B) Frequency of different household sizes in the study among households with at least two members and at least one observed infection. The study by Tibebu et al. in Ontario included 358 households with more than 8 residents, but this data was not publicly available and so those households were excluded (Tibebu et al., 2021). (C) Comparison of the observed distribution of household epidemic sizes (# infections) in the study (orange) with the average distribution of infections from forward simulation based on the model with the inferred maximum likelihood parameters (blue).

We next used our inference procedure to estimate jointly the household SAR, the variation in infectiousness ($p_{80}$), and the variation in susceptibility ($s_{80}$) from three different studies of COVID-19 spread in households (Bi et al., 2021; Dattner et al., 2021; Tibebu et al., 2021). We chose studies that included hundreds or more households with at least one infection, where there was likely to be a complete or high ascertainment rate of infections within the house, and with publicly-available data reporting the full distribution of household outbreak sizes (see Supplement for more details). These included a recruited cohort study by Bi et al. measuring seroprevalence in the city of Geneva, Switzerland between April and June 2020 (Bi et al., 2021); a household contact-tracing study with PCR testing conducted by Dattner et al. in Bnei Brak, Israel between March and April 2020 with follow up serological testing conducted between May and June 2020 (Dattner et al., 2021); and an analysis of the surveillance database for PCR-positive cases in the province Ontario, Canada conducted by Tibebu et al. between July and November 2020 (Tibebu et al., 2021)(Fig. 4). The maximum likelihood estimates and 95% confidence intervals for the inferred values of each of the three transmission parameters are reported in Table 1.

Of the three studies considered, the study conducted in Geneva had the most complete ascertainment of infection status but featured the fewest households. 2,267 households were enrolled, and only 181 had at least one seropositive individual with at least one secondary contact (i.e., the household size was greater than or equal to two). Households tended to be small. Among the households of interest, the average size was 3.0 and only 35% had four or more individuals (Fig. 4). We estimated a household SAR of 26% (95% CI 12%–37%). This was greater than the 17.3% (95% CI 13.1%–21.7%) reported in the original study though more uncertain, consistent with our results with simulated data concerning bias when heterogeneities are ignored (Fig. 3). Extreme heterogeneity in both infectiousness and susceptibility was inferred from the data, though the estimates had high uncertainty, due to the lack of mutual identifiability between parameters (Supp Figure S3). This finding is also consistent with the results from testing our inference approach on simulated data, which demonstrated that a large number of households is necessary to estimate both heterogeneities precisely.

The second dataset we analyzed was from Bnei Brak, a densely populated city in Israel, and featured many large households (637 total households were considered, 62% of which had 4 or more members; Fig. 4). Households entered the study when a symptomatic individual reported their illness to a healthcare provider and receiving a PCR-based diagnosis. Using our framework, we inferred an SAR of 40%, no infectiousness variation, and extreme susceptibility variation. However, we found that the best fit parameters failed to reproduce the observed pattern of infections (Fig. 4C). For larger household sizes, the model overpredicts both the fraction of household outbreaks with no secondary transmission and the fraction resulting in near complete infection, whereas the data show more outbreaks of intermediate size. As such, despite the fact the best estimates for the three parameters have low uncertainty, no conclusion can be drawn about the presence or amount of heterogeneity in this population.

The largest dataset we considered came from the provincial public health surveillance database of the province of Ontario, Canada, and included 28,994 households with at least one infection and at least two residents (Fig. 4B). All positive PCR tests in the province were linked back to addresses and then used to estimate the number of infections in each household. While this passive approach risks incomplete ascertainment of the infection status of individuals in a household, the widespread availability and high uptake of PCR tests in the province during the study period, especially for contacts of cases, suggest that this data may still provide a good estimate of the true household outbreak size. The maximum likelihood estimate for the household SAR was 23%. Our results also suggest the presence of substantial heterogeneity in infectiousness — with 16% of individuals being responsible for an average of 80% infections — and extreme heterogeneity in susceptibility — with 2 percent of individuals responsible for 80% of the overall susceptibility. The distribution of household infections predicted by the best fit parameters from our inference closely resembled the observed infections, suggesting our model describes the data well (Fig. 4C).

### 3.4. Application to the design of household intervention studies

Households are a useful setting to quantify the impact of interventions to reduce disease transmission, such as behavior change, personal protective equipment, vaccines, or treatment (Caleo et al., 2018; Papenburg et al., 2009; Goh et al., 2004). Since household cohorts are costly, it is useful to calculate how many participants will be needed to detect the expected effect of a given intervention reliably (i.e., to “power” the study) (Lachin, 1981). When substantial inter-individual heterogeneity is present, there is reason to believe that the sample size needed to detect an effect will be larger than predicted based on models that do not include heterogeneity. Using forward simulations and our inference framework, we analyzed the power of an imagined study of a household intervention that reduces the SAR between a control group (assumed SAR=25%) and an intervention group (assumed SAR < 25%)(Table 2). We considered three kinds of distributions of household sizes in the sample: first, all households having the same number of members (for all numbers between 2 and 8); second, households having between 2 and 8 members at the rates observed in American households as recorded by the Census Bureau (U.S. Census Bureau, 2022); and third households having between 2 and 6 members at the rates observed in Guatemalan households (a country with a large average household size) as recorded by the United Nations database of household sizes (United Nations, 2022). Note that these power calculations concern the ability to detect the presence of an effect, not the ability to ascertain the precise SAR in either the control or intervention group.

In the absence of heterogeneity, we estimated that a study of 1000 individuals divided into households of size 2 had 99% power to detect a reduction in SAR from 25% to 10% but only 59% power to detect a reduction from 25% to 15%. With a smaller study size of only 200 individuals, the powers were reduced to 79% and 39%, respectively.

We found that the amount of heterogeneity in the relative susceptibility and infectiousness of individuals in the study households had a minor effect on the power of a study to detect a change in SAR. For the study with 200 individuals described above, the power to detect a reduction from 25% to 10% was 8% points lower (79% versus 71%) in the presence of high heterogeneity in both susceptibility and infectiousness $\left(s_{80}=p_{80}=20\%\right)$ as compared to no heterogeneity. To detect a reduction in SAR from 25% to 15%, the power was 7% points lower (39% versus 32%). This relatively minor difference was consistent with the outcome of our experiments with simulated data, which showed that the SAR can be reliably inferred for any combination of heterogeneities present in the data-generating dynamics.

When comparing study designs with the same total number of individuals divided into households of size 4 versus households of size 2, we found that households of size 4 produced significantly greater power for all the tested reductions in SAR and amounts of heterogeneity. For example, to detect a reduction in SAR from 25% to 15% in the absence of heterogeneity with 1000 individuals in a study, a population divided among 250 households of size 4 provides 90% power, as compared to 59% power from the same number of individuals divided among 500 households of size 2. The relative advantage of the larger households increased as the amount of heterogeneity present increased. The larger households enjoy greater powers because the effect of heterogeneity can be more clearly separated from the role of the SAR (Fig. 2). In contrast, in households of size 2, increasing $s_{80}$ has an indistinguishable effect from decreasing SAR, which can obscure the difference in SAR between the control and the intervention arms. In our supplemental analysis, we considered populations of individuals divided among households of each size between 2 and 8, and we found that sizes between 4 and 7 each provided nearly identical powers while both size 3 and size 8 provide less power than that (but still much more power than households of size 2) (Supp Table S1).

To determine the effect that the exact distribution of sizes played in power calculations, we also compared two realistic size distributions, one roughly based on American households with size greater than 1 (average size 3.0) and the other based on Guatemalan households with size greater than 1 (average size 4.4). The same power calculations performed for groups of people divided according to these distributions indicated that power increases with average household size. The powers calculated from American household sizes were between the powers for a study composed exclusively of households of size 2 and those for a study with exclusively households of size 4. The powers calculated from Guatemalan households sizes were nearly identical to powers calculated from a sample of households of size 4. For all the studies that included households of size greater than 2, there was a greater decrease in power as heterogeneity increased compared to studies with only households of size 2.

Overall, these results demonstrate that despite the need for large sample sizes to quantify the degree of infectiousness or susceptibility variation in household studies (Fig. 4), more moderate study sizes can still be used to accurately quantify the SAR (Fig. 4) and to estimate the effect of interventions to reduce SAR (Table 2) even in the presence of these heterogeneities.

## 4. Discussion

We found that variation in the susceptibility and infectiousness of individuals has a significant impact on disease outbreaks within small, well-mixed populations such as households, and that the degree of this variation can be inferred from household transmission studies that report only the final outbreak size in each home. The inference method we developed combines exact forward simulation of any dynamic model — as opposed to existing methods that rely on simplified models with analytic solutions — with maximum likelihood estimation, and uses only modest and generally accessible computational resources. We showed that our inference approach can produce unbiased estimates of the household secondary attack risk (SAR), as well as the heterogeneity among individuals in both susceptibility to infection and transmissivity once infected. We found that when heterogeneity is present in the data-generating dynamics but unaccounted for in the parameter inference, estimates of the SAR become biased — especially as the amount of susceptibility variation increases. However, we identified several challenges in inferring inter-individual variability from household studies. When the study population contained fewer than a few thousand individuals (highly-cited household studies of COVID-19 transmission have 500–1000 individuals Li et al., 2020; Jing et al., 2020), estimates of heterogeneity had high uncertainty, and we observed that households of size two were generally uninformative about the heterogeneity present in susceptibility and infectiousness.

We made several important assumptions in our model. Like many other household transmission studies, we assumed that all households are well-mixed populations. More realistically, some individuals may have more intense contacts with others and secondary contacts of individuals known to be infected can take various levels of precaution to avoid infection. This contact heterogeneity could conflate our estimates of variation in infectiousness or susceptibility, meaning it is still possible that the inferred heterogeneity is caused by a mixture of behavior, biology, and host-pathogen interaction. Another key assumption was that there are no cases of multiple importations into households, meaning our results are most applicable to circumstances in which risk from the community is low and risk from household contacts is high. We also assumed that the distributions of relative susceptibility and infectiousness were agnostic to individuals’ biological traits such as age, sex, or prior immunity, and thus were identically and randomly distributed across individuals in all households. The high level of heterogeneity we inferred from COVID-19 household data highlights the utility of this approach to detect variation at the scale of a population even in the absence of known risk factors. Lastly, we assumed that the duration of the latent period, the duration of the infectious period, susceptibility in the population, and infectiousness in the population followed lognormal distributions. This is consistent with many empirical studies (e.g. Brooks-Pollock et al. 2020, Shrestha et al. 2022), but our results are a consequence of our choice of distribution, and we have not conducted an analysis of any biases introduced by that choice. However, because our approach allows for an arbitrary model of infection spread, each of these limitations could easily be addressed in future work.

Household infection studies must make trade-offs in recruitment method, sample size, testing methodology, and index case definition because of the constraints of time, funding, and feasibility. We applied our inference procedure to three diverse studies of household spread of SARS-CoV-2, but found that only Ontario’s vast provincial database of positive PCR tests allowed us to make reliable estimates of the individual-level variation in infectiousness and susceptibility. For the Geneva-based household serosurvey (Bi et al., 2021), we could not narrow down precise estimates of heterogeneity, likely due to the small sample size. Our tests on simulated data suggested thousands of individuals were needed for precise estimation. For the Bnei Brak study (Dattner et al., 2021), we found that even the best-fit model did not describe the data well, suggesting that assumptions of our model were violated (some possibilities are discussed below). Our results using data from Ontario underscore the indispensable role that data from broad public health surveillance plays in modeling disease spread.

Estimates from the Ontario dataset suggested moderate levels of infectiousness variation and high levels of susceptibility variation, with 16% (95% CI 14%–18%) of individuals responsible for 80% of secondary infections and 2% (95% CI 0%–4%) of individuals responsible for 80% of population-level susceptibility. An individual’s susceptibility to infection may fluctuate over time in relation to overall wellness and other factors. Our model’s prediction of a large amount of variation in susceptibility is best understood as a reflection of the time-average of susceptibility differences. While the top 2% most susceptible household members are observed to account for 80% of total susceptibility at any given moment in time, the members of that top 2% might change as time passes. The close agreement between the distribution of household outbreak sizes in the observed data and those predicted by the best-fit model gives good reason to think that the presumed model of disease spread and individual variation described the true dynamics well. Even so, we found that the discrepancy between the observed and predicted number of households with many but not all individuals infected grew as household size increased. The passive surveillance approach used in this study is unlikely to yield full infection histories in each household, which may partially explain why our model overpredicts the size of outbreaks in large households. Further work is needed to examine the possible bias introduced by incomplete ascertainment of infection status. Contrary to the hypothesis of Tibebu et al. (2021), our estimated SAR for this dataset was *higher* than the crude estimate assuming all non-index cases were infected by the primary case, even though our model allows for infections to spread over multiple generations. This difference is consistent with our finding that failing to model heterogeneities when they are present biases predictions of SAR downwards (Fig. 3B and C), which in turn highlights the importance of accounting for heterogeneity to develop a clear picture of the risk to the household contact of an infected individual.

In the case of SARS-CoV-2, age plays a role in determining susceptibility and infectiousness. In their analysis of data from Bnei Brak, Dattner et al. (2021) found strong evidence that children are less susceptible than adults. The city of Bnei Brak has a young population, with 51% of individuals under the age of 20. Our model drastically underpredicted infections in households of size 2 and overpredicted infections in large households. Declining to stratify the population by age in our inferences may have contributed to these problems with the model fit because larger households tend to contain more children and thus have a younger average age than smaller households. In a similar vein, Cauchemez et al. have shown in the context of influenza that larger households have smaller risks of infection spreading between each pair of individuals, all else being equal (Cauchemez et al., 2009). Incorporating this kind of explicit dependence on household size into our model could ameliorate the poor fit in Bnei Brak. The enrollment process and follow up condition (return visits for testing secondary contacts were only made when a household contact reported *symptoms*) may also be the source of disagreement between data and model in this setting. Since individuals in larger households are at greater risk (due to having a large number of close contacts who are themselves at risk), under-ascertainment of infection status may censor the tail of the final size distribution.

There are several possible extensions to our work that could improve the fit of the model to diverse datasets, increase the precision of estimates of heterogeneity, and incorporate other information available in some household studies. A combined approach to modeling heterogeneity could allow infectiousness or susceptibility to additionally have different average values or degrees of variance depending on individual or household risk factors, such as age group or household size. Instead of assuming a single introduction of infection to the household, we could allow a constant or time-varying rate of infections from community contacts. Some household transmission studies contain partial information about the order of infection events (from, for example, dates of symptom onset), and our inference approach could be expanded to include this data and jointly infer additional parameters, dates of infection, and transmission paths. While our current inference method relies on calculating the full likelihood function, for model expansions with more unknown parameter values, it could be adapted to instead draw samples (e.g. with a Markov-chain Monte Carlo approach) from the model likelihood (or posterior distribution).

While this work was in preparation, two other recent studies examined the degree of variation in infectiousness for COVID-19 using household data (Toth et al., 2021; Tsang et al., 2023). Toth et al. (2021) examined a cohort of households in Utah in the United States, containing approximately 30 households with two or more infections captured either by self-reported positive PCR test or a positive serological test. Using a modified chain-binomial model for final outbreak size, that accounted for individual-variation in infectiousness, they used a maximum likelihood inference method that accounted for missing infections to estimate SAR=35% and $p_{80}=15\%$. Tsang et al. (2023) fit a transmission model that allowed for variability in infectiousness to 17 different household studies from around the world in which the index case was (assumed to be) known. They simultaneously estimated infection times using an MCMC-based method to sample both infection parameters and times. Their estimates for $p_{80}$ ranged from 2% to 43%, with a pooled best estimate from a meta-regression of $p_{80}=31\%$. Their model additionally allowed the secondary attack risk to vary with household size and their model reproduced the distribution of infection sizes seen in each study well, although there were non-identifiability issues with individual model parameters. Our work is unique in allowing an explicit and general infection model, simultaneously quantifying infectiousness and susceptibility variation, and examining the impact of this variation on our ability to accurately infer household SAR and its response to interventions. Future work should combine our advances with the approaches of Toth et al. and Tsang et al. to accounting for imperfect test sensitivity and specificity, variation in SAR with household size, multiple importations, and partial knowledge of infection timing.

Our framework for estimating heterogeneity and overall rates of infection within a household is relevant not only because of what estimates imply about disease spread within a larger population, but also because of what they tell us about transmission in this vital setting for the control of epidemics. We found that even a small study can precisely estimate the SAR in the presence of heterogeneity among individuals provided that the study allows for the possibility of heterogeneity in its model of disease dynamics. Our analysis of the power of a study to detect a difference in SAR indicates that the presence of heterogeneity does not make an inference of SAR impossible. The flexibility offered by the ability to make inferences with arbitrary dynamic models of infection is additionally useful because it allows our framework to be extended in the future to many kinds of household interventions such as masking or therapeutics for SARS-CoV-2 and other respiratory pathogens.

## Supplementary Material

1

## Figures and Tables

**Table 1:** Estimating parameters of COVID-19 transmission heterogeneity from household studies.

Population	Fit with both kinds of heterogeneity			Original report
	SAR	*p*_80_	*s*_80_	SAR

Geneva, Switzerland (Bi et al., 2021)	26% [12%, 37%]	4% [2%, 80%]	18% [0%, 80%]	17%^b^ [13%, 22%]
Bnei Brak, Israel (Dattner et al., 2021)	34% [31%, 37%]	80% [64%, 80%]	2% [0%, 6%]	36%^a^ [34%, 38%]
Ontario, Canada (Tibebu et al., 2021)	23% [22%, 24%]	16% [14%, 18%]	2% [0%, 4%]	19%^a^ [19%, 20%]

Estimates for household secondary attack risk (SAR), degree of infectiousness variation (*p*_80_), and degree of susceptibility variation (*s*_80_) in three study populations (maximum likelihood estimate with 95% confidence intervals).

a SAR reported as a simple fraction of secondary contacts infected over total secondary contacts (discounting the follow up serological survey in the case of the results from Bnei Brak in Dattner et al., 2021).

b SAR reported as the best fit value from a chain binomial model with no heterogeneity.

**Table 2:** Power to measure effects of household interventions on transmission.

Household size / size distribution	Absolute SAR reduction:	0.15			0.10		
	Heterogeneity:	None	Medium	High	None	Medium	High

	# Individuals				Power		

2	200	79%	73%	71%	39%	34%	32%
	1000	99%	98%	98%	59%	48%	44%

4	200	96%	89%	84%	65%	53%	47%
	1000	>99%	>99%	>99%	90%	78%	76%

America	200	92%	86%	82%	56%	47%	46%
	1000	>99%	99%	99%	85%	77%	77%

Guatemala	200	97%	88%	83%	65%	50%	46%
	1000	>99%	>99%	>99%	85%	76%	78%

We consider a household intervention that reduces the SAR from its baseline value in Group 1 (fixed at 25%) to a lower value in Group 2 (either 15% or 10%). 2000 simulated studies were conducted for each combination of parameters. Power is calculated using a one-sided confidence interval with significance of α = 10%. In the scenario with “medium” heterogeneity, *s*_80_ = *p*_80_ = 50%. In the scenario with “high” heterogeneity, *s*_80_ = *p*_80_ = 20%. The distribution of household sizes for the American population was based on Census Bureau statistics (U.S. Census Bureau, 2022). For Guatemala, the approximation was based on the United Nations database of household composition (United Nations, 2022).
