# Supplementary material for: Quantifying individual-level heterogeneity in infectiousness and susceptibility through household studies
Source: Epidemics. Author manuscript; Available in PMC 2023 Oct 24. (PMC10594662; doi:10.1016/j.epidem.2023.100710)
Supplement: 1 [file NIHMS1929372-supplement-1.pdf]

SUPPLEMENTARY INFORMATION

Quantifying individual-level heterogeneity in infectiousness and susceptibility through household studies

Thayer L Anderson[1], Anjalika Nande[1], Carter Merenstein[2], Brinkley Raynor[3], Anisha Oommen[1,4], Brendan J Kelly[5], Michael Z Levy[3], and Alison L Hill[1,4,*]

[1]Institute for Computational Medicine, Johns Hopkins University, Baltimore, MD 21218
[2]Department of Microbiology, Perelman School of Medicine, University of Pennsylvania, Philadelphia, PA 19104
[3]Department of Biostatistics, Epidemiology, & Informatics, Perelman School of Medicine, University of Pennsylvania, Philadelphia, PA 19104
[4]Department of Biomedical Engineering, Johns Hopkins University, Baltimore, MD 21218
[5]Division of Infectious Diseases, Department of Medicine, Perelman School of Medicine, University of Pennsylvania, Philadelphia, PA 19104
[*]Correspondence to: alhill@jhmi.edu

Supplementary Methods

Modeling heterogeneity in infectiousness and susceptibility

Basic framework

Under our model, the probability that individual j is infected after an exposure to an infected contact i that lasts for the duration of their infection is:

$$p_{\text{infection},(i \to j)} = 1 - e^{-\beta \psi_j \phi_i \tau_i} \tag{S1}$$

where τ_i is the duration of the infectious period for the infected individual, ϕ_i is their relative infectiousness, ψ_j is their contact's relative susceptibility, and β is a model parameter that denotes the average probability per contact per unit time of transmitting infection.

The distribution of the relative infectiousness and susceptibility in the population can follow arbitrary single-parameter distributions with mean 1 and codomain $[0, \infty)$. For this paper, we model both distributions as lognormal random variables with mean 1 and variance Var_χ (where χ refers to the random variable ϕ or the random variable ψ). The parameters of the lognormal distribution — μ, the mean of the log of the variable, and σ, the standard deviation of the log of the variable — are then

$$\mu_\chi = -\frac{1}{2} \log \left(\text{Var}_\chi + 1 \right) \tag{S2}$$

$$\sigma_\chi^2 = \log \left(\text{Var}_\chi + 1 \right) \tag{S3}$$

Specifying heterogeneity of infectiousness in terms of expected fraction of transmission

We quantify the heterogeneity in infectiousness in terms of the distribution of expected secondary infections. In particular, we specify the heterogeneity with one parameter, p_{80}, which is defined as the (smallest) fraction of individuals responsible for 80% of expected secondary infections. This value is calculated in reference to a hypothetical population that is infinite and well-mixed — not based on the fraction of infections caused in a population divided among households, which may be equivalent to the "observed" fraction of infections as opposed to the "expected" fraction of infections. In the limit that infectiousness across the population is constant, $p_{80} = 80\%$. As p_{80} decreases, a smaller fraction of individuals is responsible for a greater fraction of infections and therefore the data is over-dispersed [76].

In order to understand how the constraint that p_{80} percent of individuals are responsible for 80% of expected secondary infections fixes the distribution of relative infectiousness, we first need to calculate the expected fraction of transmission from the most infectious individuals. To individual i in a population, we can assign an individual reproductive number $\nu_i = \beta \phi_i \tau_i n$ where n is their hypothetical number of contacts if placed in an imagined well-mixed population. This reproductive number represents the expected secondary infections caused by that individual. If ν_i were constant across the population, the offspring distribution would be a Poisson distribution that encodes the inherent stochasticity of the infection process [8]. In other words, ν_i captures only the heterogeneity in infectiousness between individuals, not the inherent variation in the number of secondary infections. Let f_ν be the probability density function (PDF) of the individual reproductive numbers of individuals. For a fixed ν_0, we call the expected fraction of transmission from individuals who are expected to cause at least ν_0 secondary infections $G_{\text{trans}}(f_\nu, \nu_0)$, and we calculate it as a ratio of the average secondary infections from the most infectious individuals to the average secondary infections from all individuals:

$$G_{\text{trans}}(\nu > \nu_0) = \frac{\int_{\nu_0}^{\infty} u f_\nu(u) \, du}{\int_0^{\infty} u f_\nu(u) \, du}. \tag{S4}$$

Let $f_{\text{infectiousness}}$ be the PDF of the relative infectiousness of individuals. Since the distribution of the length of the infectious period and the distribution of an individual's relative per-contact infectiousness (ϕ_i) are independent, $f_\nu \propto f_{\text{infectiousness}}$. If we let $\phi_0 = \nu_0 / \beta \tau_0 n$ be the relative infectiousness of an individual expected to cause ν_0 infections, we therefore have

$$G_{\text{trans}}(\nu > \nu_0) = \frac{\int_{\phi_0}^{\infty} u f_{\text{infectiousness}}(u)\, du}{\int_{0}^{\infty} u f_{\text{infectiousness}}(u)\, du}. \tag{S5}$$

But the distribution of relative infectiousness ϕ_i is constructed so that its mean value is 1, therefore the denominator is equal to 1 and

$$G_{\text{trans}}(\nu > \nu_0) = \int_{\phi_0}^{\infty} u f_{\text{infectiousness}}(u)\, du\,. \tag{S6}$$

In order to derive from Eq. (S6) the constraint on $f_{\text{infectiousness}}$, we introduce the infectiousness x_q, where q is any percentile, that splits the most infectious q individuals from the least infectious $1-q$ individuals. Let $F_{\text{infectiousness}}$ be the cumulative distribution function of $f_{\text{infectiousness}}$ then

$$F_{\text{infectiousness}}(x_q) = 1 - q. \tag{S7}$$

Then, for a given p_{80}, $x_{p_{80}}$ is the infectiousness that divides the most infectious p_{80} percent of individuals from the rest. As a result, our way of quantifying heterogeneity in relative infectiousness in terms of p_{80} boils down to the following two constraining equations:

$$F_{\text{infectiousness}}(x_{p_{80}}) = 1 - p_{80} \tag{S8}$$

$$0.8 = \int_{x_{p_{80}}}^{\infty} u f_{\text{infectiousness}}(u)\, du\,. \tag{S9}$$

For our specific choice that relative infectiousness follows a lognormal distribution then $f_{\text{infectiousness}}$ and $F_{\text{infectiousness}}$ are the PDF and CDF of Lognormal$(-\frac{1}{2}\log(\text{Var}_\phi + 1), \log(\text{Var}_\phi + 1))$ per Eqs.(S2) and (S3). We calculate the variance Var_ϕ of the lognormal random variable of relative infectiousness by simultaneously solving Eq. (S8) and Eq. (S9) using `scipy` least squares optimization.

The other commonly used measure of heterogeneity in infectiousness is k, the dispersion parameter of a negative binomial distribution with mean R_0, describing the number of secondary infections [8]. This distribution is derived from assuming relative infectiousness is gamma distributed with gamma scale factor k and mean 1, along with a fixed infectious period (e.x. [56, 72]). Dispersion parameters k can be converted to p80 values without any dependence on R_0, which makes p_{80} a useful scale-invariant measure of heterogeneity.

Specifying susceptibilities through s_{80}

As an infection spreads through a population with heterogeneous susceptibilities, the average susceptibility in the uninfected population changes over time. On average, the more susceptible individuals are infected first, which causes the average susceptibility in the remaining fraction of the population to decrease over time.

We specify the distribution of susceptibilities through the quantity s_{80}, which we define as the smallest fraction of the population that must be infected in order to reduce the total remaining susceptibility by 80% of its initial total (and therefore *to* 20% of its initial total). When there is no variation in the individual susceptibilities, $s_{80} = 0.8$. If $s_{80} = 0.2$, then the most susceptible 20% of individuals are responsible for 80% of the total susceptibility.

As before for infectiousness, let $f_{\text{susceptibility}}$ and $F_{\text{susceptibility}}$ be the PDF and CDF respectively of the distribution of relative per-contact susceptibility in the population. For a fixed value of s_{80}, let $y_{s_{80}}$ be the corresponding susceptibility that divides the top s_{80} percent of individuals from the bottom $1 - s_{80}$ percent such that $F_{\text{susceptibility}}(y_{s_{80}}) = 1 - s_{80}$. Then:

$$0.8 = 1 - \int_{0}^{y_{80}} u f_{\text{susceptibility}}(u)\, du\,. \tag{S10}$$

Note that the math here is identical to the math used to parametrize the distribution of individual infectiousness. In other words, if $s_{80} = p_{80}$, then the variance of the distribution of infectiousness is equal to the variance of the distribution of susceptibility.

Relating a specified secondary attack risk to the underlying transmission probability β

The model parameter β quantifies the probability per unit time that infection spreads between an average infectious individual (with relative infectiousness 1) an average susceptible contact (with relative susceptibility 1). In the absence of heterogeneity or variable infection lengths, β's value is defined by the relationship $p_{\text{escape}} = e^{-\beta\tau}$.

While performing inferences, we choose to specify the household secondary attack risk as a model input and derive the value of β that achieves that household secondary attack risk (SAR). We define the SAR as the probability of infection per household contact caused by a single initial introduction of the pathogen (i.e., not taking into account chains of infection beyond the initial → secondary). SAR can be calculated as follows:

$$\text{SAR} = 1 - \langle p_{\text{escape}} \rangle. \tag{S11}$$

We choose to perform inferences with respect to SAR because it is often the quantity of interest in empirical studies. SAR can be roughly estimated using statistical averages without the aid of a dynamic model or forward simulation, and it conveys relevant public health information: the risk to secondary contacts from a single infection in a household.

Let $H = \tau\phi\psi$ be a random variable that is the product of the random variables that describe the distribution of infection periods τ, the distribution of infectiousness ϕ, and the distribution of susceptibility ψ, and let h be its PDF. In other words, H is the effective period of infection. By analogy to the relationship $p_{\text{escape}} = e^{-\beta\tau}$, we have

$$\text{SAR} = 1 - \int_0^\infty e^{-\beta x} h(x)\, dx\,. \tag{S12}$$

Because we use lognormal random variables to describe individual susceptibility, individual infectiousness, and the duration of the infectious state, and the product of lognormal random variables is another lognormal random variable, it follows that $H = \text{Lognormal}(\mu_\tau + \mu_\psi + \mu_\phi, \sigma_\tau^2 + \sigma_\psi^2 + \sigma_\phi^2)$.

We specify SAR and the distributions of state lengths, infectivities, and susceptibilities, and we use them to calculate the model parameter β. We can evaluate the integral in question numerically in order to solve for β that produces the desired SAR.

Computational methods

Stochastic forward simulation was conducted in each household by performing an exact forward simulation based on the Gillespie method [95], modified to allow the duration of the exposed and infectious states to follow an arbitrary distribution (rather than only an exponential distribution). The code was written in Python and used the 'torch' package to implement fast calculations on the GPU and to sample pseudorandom numbers.

For each household size, simulation was conducted at each point in a three-dimensional grid of parameters. This regular grid was composed of all combinations of three parameters from the following ranges:

$$s_{80} \in [0.02, 0.04, 0.06, \ldots, 0.80] (40 \text{ values total})$$
$$p_{80} \in [0.02, 0.04, 0.06, \ldots, 0.80] (40 \text{ values total})$$
$$\text{SAR} \in [0.01, 0.02, \ldots, 0.60] (60 \text{ values total}).$$

At all points, we simulated 200,000 households of each size. This data was used in assessing the likelihood of parameter values (and therefore the maximum likelihood estimate) for simulated data (Figure 3) for empirical data (Figure 4 and Table 1) and for power calculations for household interventions (Table 2)

Dependence of reliability of inference on the distribution of household sizes

We noted that reliable inference of s_{80}, p_{80} and SAR is possible with a sufficient sample size (Figure 3). This result was based on simulated data generated by simulating household infections for individuals divided among households of size ≥ 2 is accordance with data from the US census (average size of 3.0 individuals).

We repeated this analysis for a variety of different household size distributions (Supp Figure S1), and we found that the precision and reliability of the fit generally increases with a larger average household size. Estimates of SAR are not particularly sensitive to this effect, consistent with our result that SAR estimates are precise for a wide range of sample sizes. For estimates of s_{80} there is a pronounced effect where larger average household sizes correspond to much better estimates of the amount of susceptibility variation (regardless of the kinds and amount of heterogeneity present in the data-generating dynamics). For estimates of p_{80} the distribution of individuals among households of size 3,4, and 5 (average size: 3.83) produced the most precise estimates, with the populations with smaller and larger average household sizes both yielding a greater amount of uncertainty in the estimates of p_{80}.

For the population based on American households, we found bias in the estimates of SAR for a model that discounts heterogeneity only when there was substantial variation in susceptibility (Figure 3A). When considering the affect of average household size, we found that a larger average household size produces an increasing bias in the estimate for SAR when variation in infectiousness is present but discounted by the model used for inferring SAR (Supp Figure S1 top right). For the simulated population with average size 2.77, the estimate for a SAR with a misspecified model was centered on 19%. For the population with average size 3.83, the estimate was centered on 18%. For the population with average size 4.86, the estimate was centered on 17%. This bias increases with average household size because the infection risk to each secondary contact of an infected individual is correlated with that individual's infectiousness. This behavior causes the misspecified model to depart more from the actual dynamics when infectiousness varies as household size increases.

COVID-19 household data

Numerous household studies of the spread of COVID-19 have been conducted since the pandemic first spread across the globe in 2020 [96–98]. Three major sets of factors distinguish different studies. The first set is the content of the sample — including the methodology for entering individuals into the study, the number of individuals enrolled, and how those individuals are divided among households — which plays a role in determining possible sources of error, factors that may need to be controlled for, and what inferences are possible. The second set is the timing with respect to the waves of epidemic: the probability of importation (and subsequent multiple importation) depends on the overall level of spread in the population and affects the validity of a model that assumes there is a single index case within each household [99]. The third set is the means of ascertaining the infection status of individuals and whether that test was applied to all individuals, only symptomatic individuals, or a mix of symptomatic individuals and consenting asymptomatic individuals. Most household studies of COVID-19 either ascertain the infection status of individuals by testing all contacts regardless of symptoms via reverse transcription polymerase chain reaction (RT-PCR) at least once and typically twice or more [97] or ascertain the infection history of individuals through serological testing.

We focus only on a single measure of infection from each household - the final number of individuals who were ever infected in the household (final "attack rate" or epidemic size). We assume no further longitudinal information is available, such as about the identity of the index case, or the timing of infection (or symptom onset) for any of the secondary cases. This is the most commonly studied household infection data type, as traditionally, the infection status of household cohorts was assessed retrospectively via serology, which provides very limited temporal resolution for short-term, rapidly transmitting infections. Final epidemic size is the outcome that chain binomial models were designed to describe, and so by using only this outcome we can more directly compare our method to the most common analytic approach for making inference on transmission parameters from household studies. However, many household studies are longitudinal, and may include diagnoses obtained with molecular or antigenic tests, or with symptom-based reporting. Thus, they may include additional information that could inform the transmission chain - such as timing of first positive test result or symptom onset. This extra data is always imperfect and study specific (e.g. imperfect test sensitivity or specificity, measurements only every few days, delay from infection to symptoms, lack of genetic data to identify household vs community acquisition), and so more complex models of the data generation process are required to compare underlying infection models to data. Thus, to minimize our model parameters and allow inference from multiple distinct studies, we have focused only on final epidemic size.

Based on our model, an ideal study would have a large sample of households with three or more individuals, be conducted during a period of low community risk, and enter households into the study randomly to avoid observation bias of symptomatic individuals, and fully ascertain the seropositivity of individuals regardless of symptoms. Due to many practical constraints, such studies are rare. We reviewed approximately twenty-five household studies and selected three studies for further analysis based on their diverse set of characteristics and their efforts to approximate these ideal circumstances. We located studies by searching for key terms such as "household transmission," "attack rate," and "secondary infections" between June and September of 2021. For the majority of studies that we excluded, we excluded them on the grounds that they had fewer than two hundred total positive cases, which initial theoretical investigations revealed to be insufficient to make precise estimates of our three parameters of interest. Another discriminant among studies was the availability of data. Some studies that relied on passive surveillance databases, such as the UK's HOSTED database did not provide open access to their data tabulated by household size and were therefore unsuitable for our purposes. We preferred studies of households from the initial wave of the pandemic in 2020, as such studies avoided the complications of prior immunity and variant emergence. Since no perfect studies for our purposes existed, we made compromises that prioritized having a diverse range of studies included in our analysis in hopes of gaining more insight into what qualities of study made it tractable for our analysis.

All three household studies that we subjected to analysis were drawn from a time period where ancestral strains of COVID-19 predominated. The three studies display a wide range of methodologies for ascertaining infection status/history:

Geneva, Switzerland

The study of seroprevalence in Geneva, Switzerland conducted by Bi et al. between April and June 2020 [71] was based on a recruited serosurvey by Stringhini et al. with participants randomly drawn and recruited from past population-representative household surveys in Geneva [93]. Each participant had their blood drawn and tested for anti-SARS-CoV-2-IgG antibodies exactly once.

Bnei Brak, Israel

The household contact-tracing study conducted by Dattner et al. in Bnei Brak, Israel was based on data from Israel's PCR testing database collected in Bnei Brak between March and April 2020. Initial visits were made to households when an individual reported symptoms of SARS-CoV-2. Later visits were conducted to re-test previously positive individuals or to test newly symptomatic individuals.During visits, "additional tests within a household were conducted, even if no symptoms were observed" [78]. Afterwards, between May and June 2020, a follow-up serological survey was conducted for all household members over the age of six in a fraction of the households where testing had taken place [78]. Households were included in the serological survey if the household members included up to four adults and included at least two individuals who tested negative via PCR. This serological survey was used to estimate the rate at which infections were not detected. We applied the same methodology as the study authors to create 20 datasets in which PCR-negative individuals were, with a random chance provided by the study authors, assigned to positive status for the purposes of analysis in our final size data. Although this study includes a proxy for initial symptom onset, for all these we considered only final size data in our model, as this is the most generally available data from household studies, and we hope for our methods to be widely adopted.

This study initially ascertained infection status by RT-PCR testing of symptomatic individuals and consenting asymptomatic individuals but conducted a follow up serostudy to estimate the false negative rate [78]. We applied a randomized correction to the reported number of infections in each household by generating 20 studies where individuals reported as negative were considered positive at random with probability given by the reported correction factor of the study authors.

Ontario, Canada

The study conducted in the province of Ontario, Canada by Tibebu et al. between July and November 2020 was based on a surveillance database of PCR-positive cases in the province [79]. Households were linked by matching residential addresses, and number of household contacts was determined by reported household

size. 358 households (1.2% of all households) with 9 or more individuals were included. These households were pooled in the reported data in such a way that a household of size 9 with 4 secondary infections (4/8 secondary contacts infected) was indistinguishable from a household of size 13 with 6 infections. We excluded this small fraction of households and restricted our analysis to households with between 2 and 8 individuals.

Contours of likelihood for household studies

We used our framework to infer SAR, s_{80}, and p_{80} in the context of three household studies: one conducted in Bnei Brak, Israel; one conducted in Geneva, Switzerland; and one conducted in Ontario, Canada. In addition to calculating a maximum likelihood estimate (MLE) and 95% credible intervals, we also calculated a three-dimensional likelihood surface (equivalently, a probability density of the posterior with uniform priors) for those parameters.

This surface can encode valuable insights even when a precise estimate of one or more parameters is not possible. For the study conducted in Geneva, the 95% confidence intervals for the estimates s_{80} and p_{80} showed extremely high uncertainty in the MLE for these parameters. The contours of the likelihood surface (Supp Figure S3) indicate that this uncertainty is due to a steep trade-off between the parameters that govern heterogeneity (s_{80} and p_{80}) and the parameter that governs average risk to secondary contacts SAR.

For the studies conducted in Bnei Brak and in Ontario, the probability density was localized almost entirely to a single point on the discrete grid of points where the likelihood was calculated (Supp Figure S2 and Supp Figure S4). The lack of trade offs between the parameters and the lack of uncertainty is due to two factors. First and most importantly, these two studies featured many times more households (Figure 4C) than the study in Geneva. More households in the sample steepens the multinomial likelihood (Equation 1): an equal deviation between the observed and expected frequencies of infection for a given set of parameters θ produces a lower likelihood if there are more households included the observation. Second, these two studies also featured more many-person households than the study in Geneva, which is known to correspond to greater certainty in the estimates of parameter values (Figure 3). That both the Bnei Brak study and the Ontario study produced localized contours but the model of best fit for Bnei Brak failed to reproduce the observed infections underscores the importance of judging the fit of a model in absolute terms and not only based on the narrowness of the 95% CI.

Supplementary References

[94] Kremer C, Torneri A, Boesmans S, Meuwissen H, Verdonschot S, Vanden Driessche K, et al. Quantifying superspreading for COVID-19 using Poisson mixture distributions. Scientific Reports. 2021;11(1):14107. doi:10.1038/s41598-021-93578-x.

[95] Gillespie DT. Exact stochastic simulation of coupled chemical reactions;81(25):2340–2361.

[96] Madewell ZJ, Yang Y, Longini IM Jr, Halloran ME, Dean NE. Household Transmission of SARS-CoV-2: A Systematic Review and Meta-analysis. JAMA Network Open. 2020;3(12):e2031756. doi:10.1001/jamanetworkopen.2020.31756.

[97] Fung HF, Martinez L, Alarid-Escudero F, Salomon JA, Studdert DM, Andrews JR, et al. The Household Secondary Attack Rate of Severe Acute Respiratory Syndrome Coronavirus 2 (SARS-CoV-2): A Rapid Review. Clinical Infectious Diseases. 2021;73(Supplement_2):S138–S145. doi:10.1093/cid/ciaa1558.

[98] Madewell ZJ, Yang Y, Longini IM Jr, Halloran ME, Dean NE. Household Secondary Attack Rates of SARS-CoV-2 by Variant and Vaccination Status: An Updated Systematic Review and Meta-analysis. JAMA Network Open. 2022;5(4):e229317. doi:10.1001/jamanetworkopen.2022.9317.

[99] Longini IM Jr, Koopman JS, Monto AS, Fox JP. Estimating household and community transmission parameters for influenza. American Journal of Epidemiology. 1982;115(5):736–751. doi:10.1093/oxfordjournals.aje.a113356.

[100] Dattner I, Goldberg Y, Katriel G, Yaari R, Gal N, Miron Y, et al. The role of children in the spread of COVID-19: Using household data from Bnei Brak, Israel, to estimate the relative susceptibility and infectivity of children. PLOS Computational Biology. 2021;17(2):e1008559. doi:10.1371/journal.pcbi.1008559.

Supplementary Figures

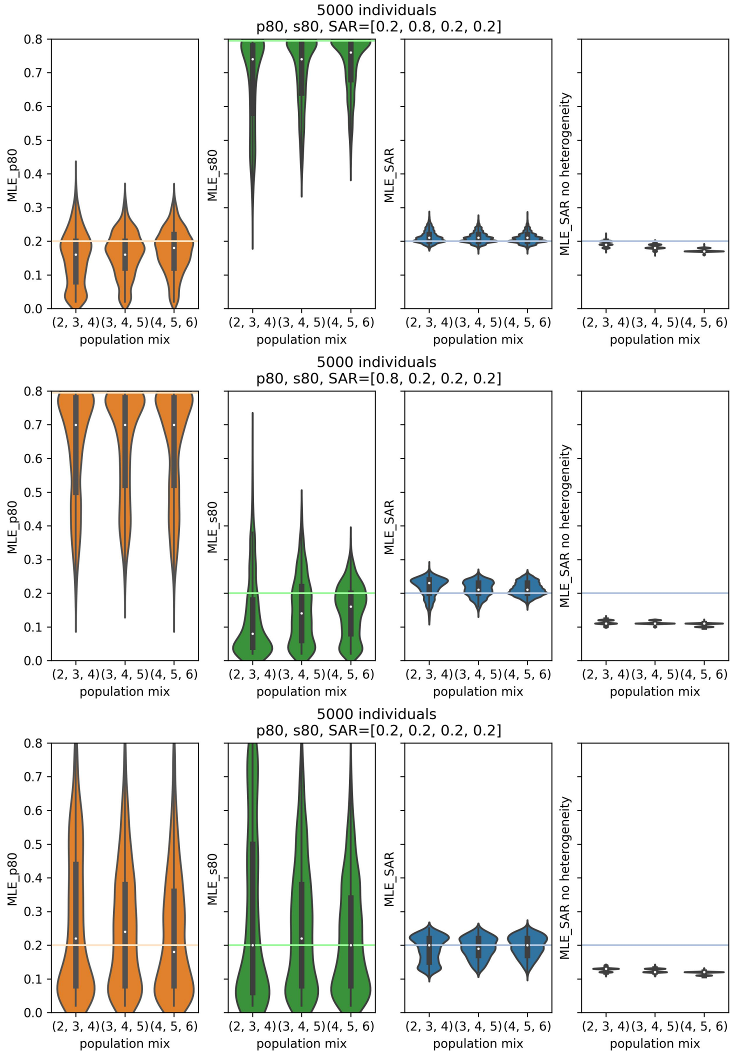

Figure S1: **Maximum likelihood estimates for SAR and degree of heterogeneity on simulated data based on the sizes of included households.** Simulated datasets were generated using 5,000 individuals divided evenly among households of three different sizes. In each plot, the leftmost violin represnts the distribution of predictions when individuals are divided evenly among households of size 2, 3, and 4 (average size: 2.77). The center: 3, 4, and 5 (average size: 3.83). The right: 4, 5, and 6 (average size 4.86). A) Simulated data includes only variation in infectiousness (p_{80}). B) Simulated data includes only variation in susceptibility (s_{80}). C) Simulated data includes variation in both infectiousness and susceptibility. The three left panels show the distribution of maximum likelihood estimates (MLEs) for the three parameters of interest - the secondary attack risk (SAR), the variation in infectiousness (p_{80}), and the variation in susceptibility (s_{80}). For each panel, the colored horizontal line is the actual value of the parameter used to generate the simulated data, and the white dot is the mean of the individual MLEs. The rightmost panel shows the MLE for the SAR if heterogeneities are not included in the model inference framework. Parameters were estimated for 1,500 different simulated datasets. Other model parameters were fixed as described in the Methods.

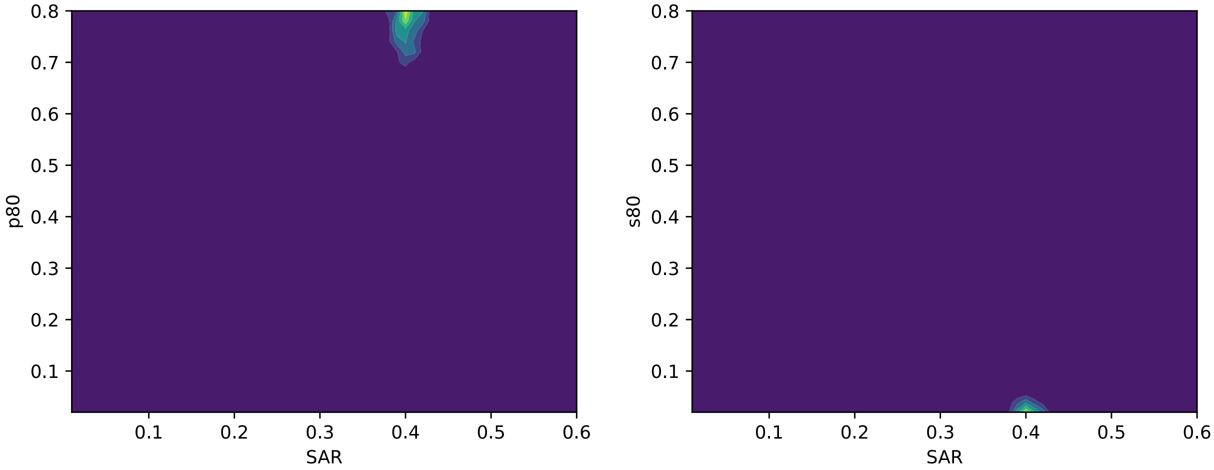

Figure S2: Likelihood surfaces describing the probability of the model parameters given the observation of the study conducted in Bnei Brak, Israel by Dattner et al. [78]. Yellow is more likely, blue is less likely. Left: likelihood plotted as a function of SAR and p_{80}. Right: likelihood plotted as a function of SAR and s_{80}. Each two dimensional surface marginalizes over the third, not shown, parameter.

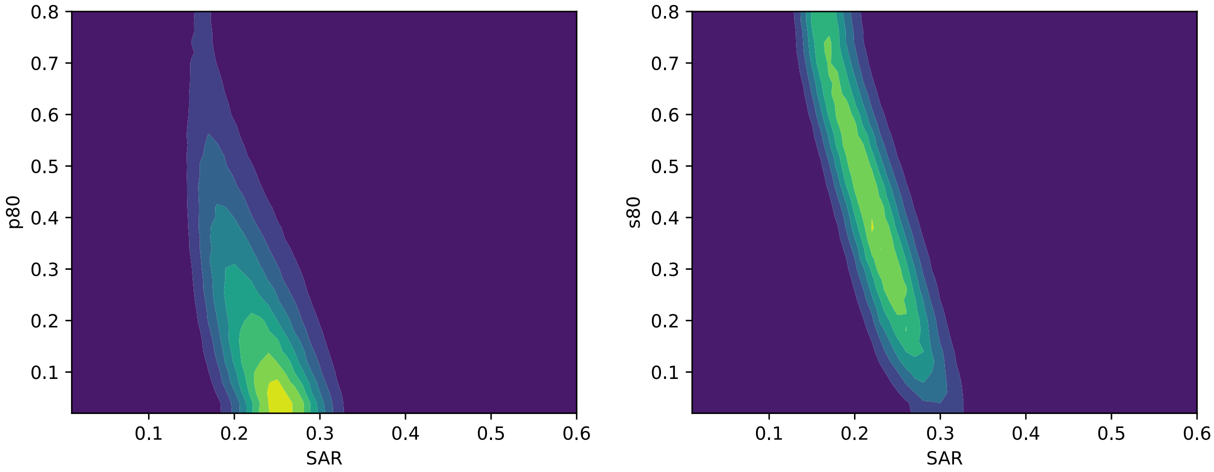

Figure S3: Likelihood surfaces describing the probability of the model parameters given the observation of the study conducted in Geneva, Switzerland by Bi et al. [71]. Yellow is more likely, blue is less likely. Left: likelihood plotted as a function of SAR and p_{80}. Right: likelihood plotted as a function of SAR and s_{80}. Each two dimensional surface marginalizes over the third, not shown, parameter.

	Absolute SAR reduction:	0.15			0.10		
	Heterogeneity:	None	Medium	High	None	Medium	High
Household size / size distribution	# Individuals	Power					
2	200	79%	73%	71%	39%	34%	32%
	1000	99%	98%	98%	59%	48%	44%
3	200	93%	87%	83%	55%	49%	46%
	1000	>99%	>99%	>99%	79%	73%	74%
4	200	96%	89%	84%	65%	53%	47%
	1000	>99%	>99%	>99%	90%	78%	76%
5	200	98%	90%	85%	70%	54%	47%
	1000	>99%	>99%	>99%	93%	77%	75%
6	200	98%	90%	82%	65%	50%	45%
	1000	>99%	>99%	>99%	89%	75%	72%
7	200	99%	90%	82%	63%	46%	44%
	1000	>99%	>99%	>99%	87%	67%	70%
8	200	98%	86%	80%	53%	41%	38%
	1000	>99%	>99%	99%	79%	59%	62%

Table S1: **Power to measure effects of household interventions on transmission**. As in Table 2, we consider a household intervention that reduces the SAR from its baseline value in Group 1 (fixed at 25%) to a lower value in Group 2 (either 15% or 10%). 2000 simulated studies were conducted for each combination of parameters. Power is calculated using a one-sided confidence interval with significance of $\alpha = 10\%$. In the scenario with "medium" heterogeneity, $s_{80} = p_{80} = 50\%$. In the scenario with "high" heterogeneity, $s_{80} = p_{80} = 20\%$. Simulations demonstrate that households of size greater than 2 significantly outperform households of size 2 for powering studies to detect a reduction in SAR. Households of sizes between 4 and 8, perform about equally for all tested reductions in SAR and combinations of heterogeneity, with size 5 producing the greatest powers by a slim margin. Households of size 3 and size 8 underperform relative to households of sizes between 4 and 7, but still provide greater power than households of size 2.

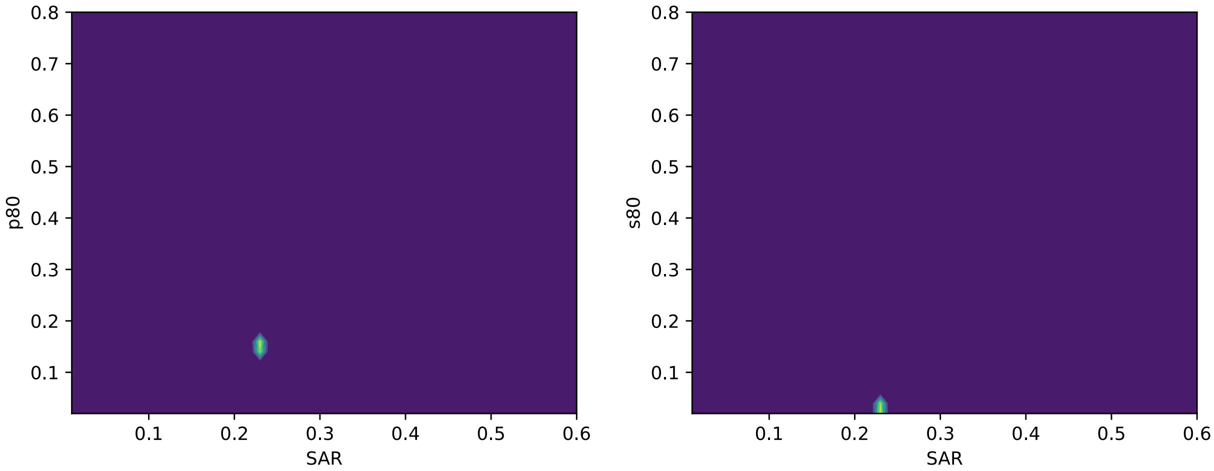

Figure S4: Likelihood surfaces describing the probability of the model parameters given the observation of the study conducted in Ontario, Canada by Tibebu et al. [79]. Yellow is more likely, blue is less likely. Left: likelihood plotted as a function of SAR and p_{80}. Right: likelihood plotted as a function of SAR and s_{80}. Each two dimensional surface marginalizes over the third, not shown, parameter.
